# Evaluation on the possibility of sound conduction independent of tympanic air cavity for severe tympanic adhesion patients by finite element analysis

**DOI:** 10.3389/fbioe.2023.1212303

**Published:** 2023-10-31

**Authors:** Xiao Qin, Yue Yin, Huiying Sun, Guodong Feng, Zhiqiang Gao

**Affiliations:** ^1^ Department of Otolaryngology, Peking Union Medical College Hospital, Chinese Academy of Medical Sciences, Beijing, China; ^2^ Medical Science Research Center, Peking Union Medical College Hospital, Chinese Academy of Medical Sciences, Beijing, China

**Keywords:** conductive hearing loss, hearing reconstruction, tympanic air cavity, tympanic vibrating material, finite element analysis

## Abstract

**Background:** For patients with severe tympanic adhesion, reconstructing the tympanic air cavity is often challenging, resulting in poor hearing reconstruction outcomes. Therefore, establishing a sound conduction pathway independent of the tympanic air cavity may be a viable method for reconstructing hearing in these patients.

**Purpose:** The objective of this study was to evaluate the feasibility of sound conduction independent of the tympanic air cavity (i.e., replacing the original cavity with a tympanic vibrating material) using finite element analysis.

**Methods:** We established a sound-structure coupling finite element model of the tympanum vibration conduction system, which included the tympanic membrane (TM), ossicular prosthesis, and tympanic vibrating material. This model was used to simulate middle ear vibrations under sound pressure, and we extracted the frequency response curve of the ossicular prosthesis’ vibration displacement amplitude to evaluate the sound conduction effect of the middle ear. Next, we adjusted the structural and mechanical parameters of the tympanic vibrating material to analyze its impact on the sound conduction effect of the middle ear. Finally, we compared the frequency response curve of the stapes footplate in normal subjects to evaluate the feasibility of sound conduction independent of the tympanic air cavity.

**Results:** The Shell tympanic vibrating material had a better vibration conduction effect compared to solid or porous tympanic vibrating material. The vibration amplitude decreases with the increasing elastic modulus of the tympanic vibrating material. Implantation of 40 kPa-shell tympanic vibrating material had the lowest hearing loss less than 5 dB, and the hearing loss with 1 MPa-porous tympanic vibrating material was largest and less than 25 dB.

**Conclusion:** Our study suggests that replacing the tympanic air cavity with a tympanic vibrating material is feasible. The establishment of a sound conduction pathway independent of the tympanic air cavity could potentially provide a method for hearing reconstruction in patients with severe tympanic adhesion.

## 1 Introduction

Hearing loss is one of the leading causes of disability worldwide. According to estimates from the World Health Organization (WHO), more than 1.5 billion people are affected by hearing loss, with 430 million experiencing moderate or severe levels. By 2050, it is projected that nearly 2.5 billion people will live with some degree of hearing loss (WHO, 2021). The incidence rate of conductive hearing loss caused by disorders of the middle ear, such as chronic suppurative otitis media (WHO, 2017) and cholesteatoma otitis media (Jennings et al., 2018), is approximately 4.86 per thousand or 55 million people worldwide. Therefore, solving the issue of hearing reconstruction in patients with tympanic adhesion is of great clinical significance.

Tympanoplasty is the standard procedure for treating these patients (Fisch, 1994; Bayram et al., 2020; Lubianca Neto et al., 2022; Gutierrez et al., 2023; Zhao and Chen, 2023). It involves removing lesions and reconstructing the middle ear structure to improve hearing. However, severe tympanic adhesion can make it difficult to restore the tympanic air cavity, affecting ossicular chain vibration and hindering successful hearing reconstruction (Neudert and Zahnert, 2017). While researchers have attempted to improve results with anti-adhesion material implantation (Deng et al., 2018; Yamamoto et al., 2021), eustachian tube dilation (Si et al., 2019), and other methods, some patients may still experience incomplete clearance of tympanic adhesion, making anti-adhesion material implantation and eustachian tube dilation ineffective for hearing reconstruction. Clinicians’ persistent concern is how to reconstruct hearing in such patients without restoring the tympanic air cavity. Currently, bone-guided hearing aids (e.g., BAHA) are the most widely used method for hearing reconstruction, as they drive vibrating parts in the ear or cochlea through direct vibration. However, these aids are expensive and have limitations such as sound distortion, MRI incompatibility, and battery replacement requirements, which can inconvenience patients (Kruyt, 2020). Therefore, finding a more convenient and effective solution for hearing reconstruction in patients with severe tympanic adhesion is of great significance.

**FIGURE 1:**
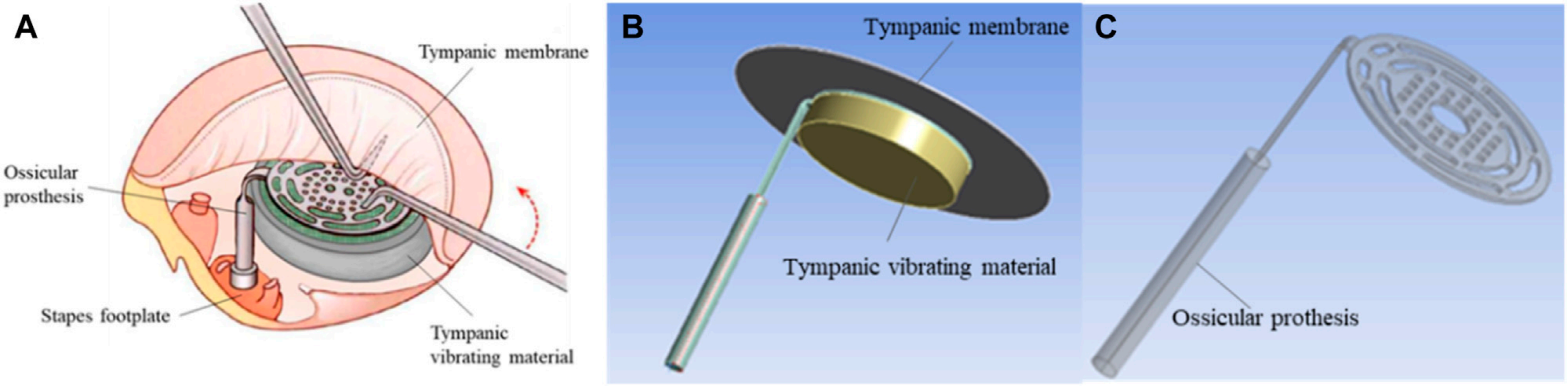
Geometrical model of the tympanic vibration conduction system. **(A)** The schematic diagram of tympanic prosthesis implantation; **(B)** The whole geometrical model of the tympanic vibration conduction system, the tympanic membrane was modeled as a circular membrane with a radius of 10 mm and thickness of 60 μm; **(C)** The geometrical model of the TORP artificial auditory ossicle.

In the air conduction pathway of sound, sound waves cause vibrations in the air, which are then transmitted through the external auditory canal to the tympanic membrane. The membrane vibrates, and this vibration is transmitted to the inner ear via the auditory ossicles (Ayerbe et al., 1999). However, in patients with tympanic adhesion, effusion, and adhesive tissue inhibit the normal vibration of both the tympanic membrane and the auditory ossicles, resulting in conductive hearing loss. Theoretically, if we can find a material that can replace the middle ear effusion or adhesive tissue in the tympanic cavity and reduce the vibration resistance of the tympanic membrane and auditory ossicles compared to adhesive tissue, we may effectively improve the hearing of patients with tympanic adhesion. Based on this assumption, the tympanic vibrating material can be implanted in the tympanic cavity working as a support material and providing vibration space for the ossicle and TM for severe tympanic adhesion patients (Figure 1A). The TM, ossicles, and tympanic vibrating material vibrate under sound pressure. Certainly, the most ideal vibrating material is air. For patients with severe tympanic adhesion, as long as the vibration amplitude of the upper surface of the tympanic vibration material under the sound can provide enough space for the vibration of the ossicle (the vibration amplitude of the ossicle in the tympanic vibration material was larger than its vibration amplitude in effusion or adhesive tissue), the reconstruction of middle ear sound conduction can be achieved. So, the key is to find the appropriate structure and material characteristics of the tympanic vibration material. As few studies have been conducted on this topic, it is necessary to first evaluate the possibility of sound conduction independent of the tympanic air cavity for severe tympanic adhesion patients.

The effectiveness of hearing reconstruction is typically assessed by measuring the vibration curve of the stapes footplate under specific sound pressure (ASTM, 2014). Although Laser Doppler Vibrometer (LDV) can be used to measure the vibration of the stapes footplate at present (Warnholtz et al., 2021), this method is invasive and usually only used to measure vibrations in human or animal cadaveric temporal bones (Ding et al., 2016). The finite element method is a tool for computing approximate solutions to complex mathematical problems. It is increasingly being used in the evaluation of middle ear vibration (Lobato et al., 2018; Zhou et al., 2019). As mentioned above, the key to sound conduction independent of the tympanic air cavity is to find the appropriate structure and material characteristics of the tympanic vibration material. The finite element method is a cost-effective and feasible alternative for middle ear vibration before LDV measurements or animal experimental verification, as it allows for the calculation of various working conditions using the same model. Harmonic response analysis is the analysis of the steady-state response of a structure to a simple harmonic load that varies sine or cosine over time, which can be used for analyzing the frequency response of the middle ear under sound pressure. Therefore, we used harmonic acoustics finite element simulation to evaluate the possibility of sound conduction independent of the tympanic air cavity.

In this study, we developed a sound-structure coupling harmonic acoustics finite element model of middle ear vibration that included the tympanic membrane (TM), ossicular prosthesis, and tympanic vibration material. This model was used to simulate middle ear vibrations under sound pressure, and the frequency response (vibration amplitude-frequency curve) of the ossicular prosthesis was extracted to evaluate the sound conduction effect in the middle ear. We adjusted the structural and mechanical parameters of the tympanic vibrating material to analyze its impact on the sound conduction effect of the middle ear. In the actual processing process, solid model processing is relatively simple. The shell structure based on the same material can provide enough vibration space for TM and ossicle due to the air cavities in it, however, the thin shell may have weaker support. The porous structure can provide relatively large vibration space while also providing strong support, however, its processing technology is relatively complex. So the solid structure, porous structure, and shell structure were designed in this study to evaluate the middle ear vibration under tympanic vibration material implantation. By comparing our results to the vibration curve of the stapes footplate in air and water, we were able to evaluate the possibility of sound conduction independent of the tympanic air cavity. These findings may provide a potential hearing reconstruction method for patients with tympanic adhesion.

## 2 Materials and methods

### 2.1 Geometrical model construction of the tympanic vibration conduction system

The schematic diagram of tympanic prosthesis implantation is shown in Figure 1. For patients with tympanic adhesions, the tympanic vibrating material was designed and implanted to prevent the formation of adhesive tissue. Besides, the mechanical properties of the tympanic vibrating material can ensure the vibration of the ossicle chain (Figure 1A). To verify the functionality of the tympanic vibrating material, a simplified geometrical model of the tympanic vibration conduction system (Figure 1B) was developed which simplified the system into three components: the tympanic membrane, a Total Ossicle Replacement Prosthesis (TORP, Figure 1C), and a tympanic vibrating material. The tympanic membrane was modeled as a circular membrane with a radius of 10 mm and a thickness of 60 μm. The TORP artificial auditory ossicle was used to replace the natural ossicles (see Figure 1B). Three different structures of tympanic vibrating material (see Table 1) were designed, including the solid cylindrical material, the porous tympanic vibrating material and the shell tympanic vibrating material. The fluid mesh was employed with a maximum element size of 0.1 mm. The geometric model of the TORP prosthesis was provided by Suzhou Jenitek Medical Co., Ltd. (Jiangsu, China).

**TABLE 1:** Comparison of three tympanic vibration materials.

	Diagrammatic graph	Size
Solid tympanic vibration material	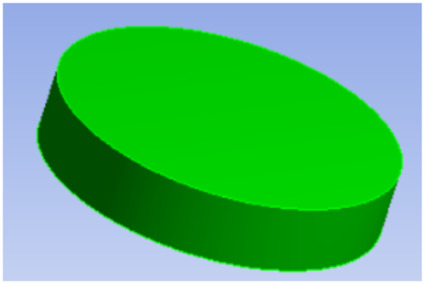	Total radius: 2.5 mm
		Total thickness: 1 mm
Porous tympanic vibration material	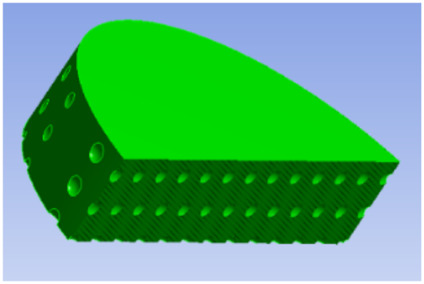	Total radius: 2.5 mm
		Total thickness: 1 mm
		Diameter of the hole: 0.2 mm
		Distance between holes: 0.4 mm
Shell tympanic vibration material	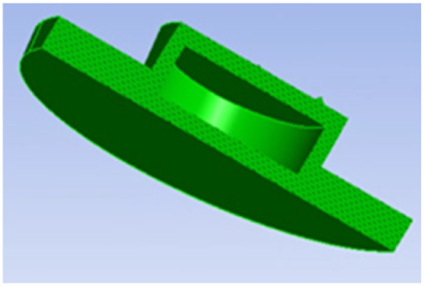	Radius of the upper surface: 1.2 mm
		Radius of the bottom surface: 2.5 mm
		Total thickness: 1 mm
		Diameter of the cylindrical cavity:2 mm
		Height of the cylindrical cavity: 0.5 mm
		Thickness of the shell: 0.2 mm

### 2.2 Sound-structure coupling finite element simulation of the tympanum vibration conduction

**FIGURE 2:**
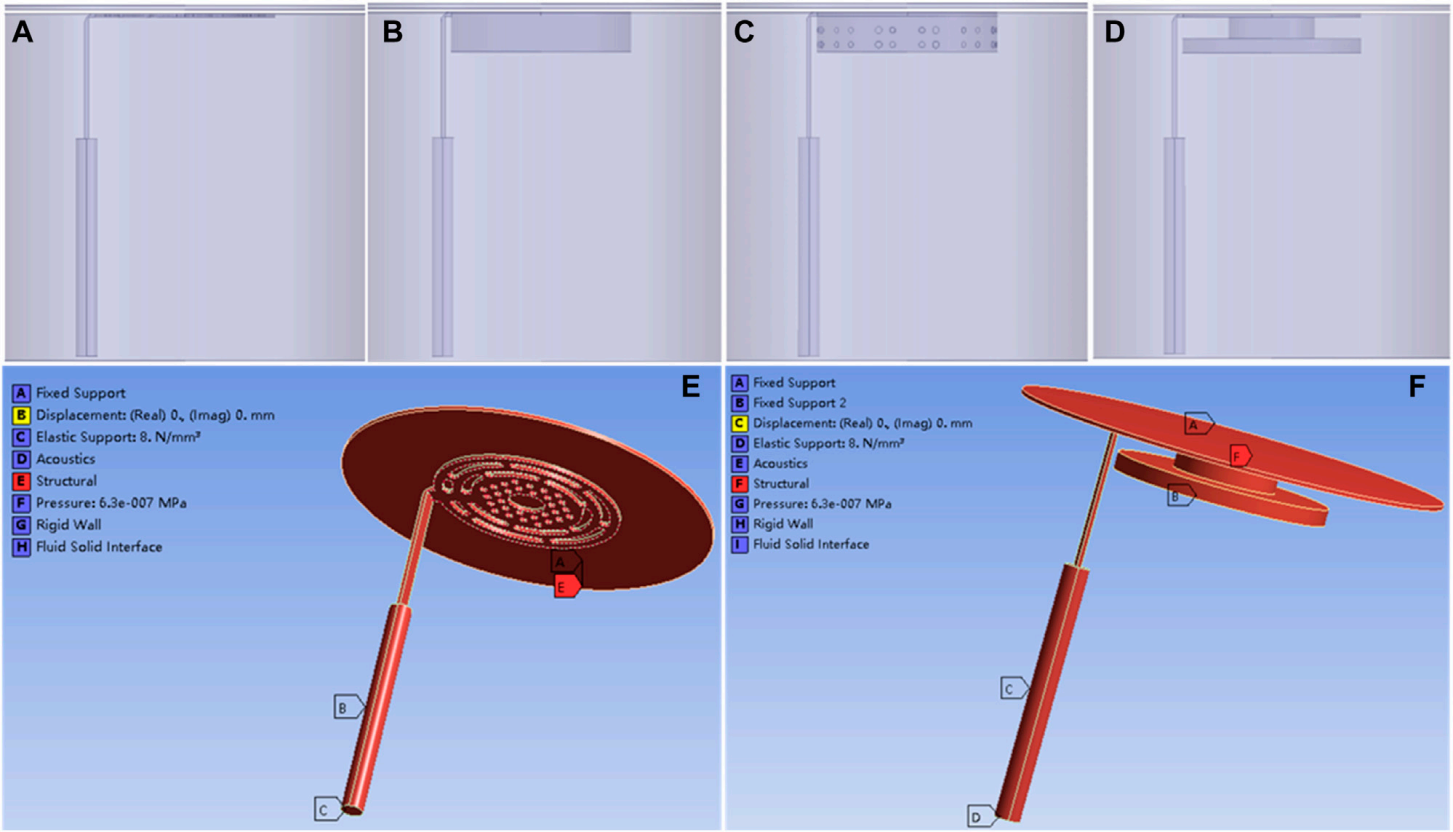
Harmonic acoustics finite element model of the tympanum vibration conduction. **(A)** acoustic domain of the model without tympanic vibrating material; **(B)** Acoustic domain of the model with solid tympanic vibrating material; **(C)** Acoustic domain of the model with porous tympanic vibrating material; **(D)** Acoustic domain of the model with shell tympanic vibrating material; **(E)** The model setup without tympanic vibrating material; **(F)** The model setup with shell tympanic vibrating material.

The finite element method is a cost-effective and feasible alternative for middle ear vibration before LDV measurements or animal experimental verification, as it allows for the calculation of various working conditions using the same model. It is increasingly used in the evaluation of middle ear vibration. Harmonic response analysis is the analysis of the steady-state response of a structure to a simple harmonic load that varies sine or cosine over time, which can be used for analyzing the frequency response of the middle ear under sound pressure. Therefore, we used harmonic acoustics finite element simulation to evaluate the possibility of sound conduction independent of the tympanic air cavity. According to the air conduction pathway of sound, sound causes vibration in the air, which drives the vibration of the TM and ossicles. A finite element simulation of tympanum vibration conduction was conducted using a harmonic acoustics model (Figure 2) on Ansys Workbench 2019R1. The physical regions included the TM, artificial auditory ossicle, and tympanic vibrating material. The acoustic region was defined as an enclosed cylinder generated from the tympanic membrane, TORP, and tympanic vibrating material with a radius of 5 mm, filled with air (Figures 2A–D). The external sound source is received by the auricle and ear canal. Sound waves cause vibrations in the air, which are then transmitted through the external auditory canal to the TM. This was simplified as a 0.63Pa (90 dB) uniform sound pressure boundary on the upper surface of the air sound domain with frequency ranging from 100 Hz to 10 kHz. The side and bottom surfaces of the acoustic domain were set as rigid walls to simulate the effect of the tympanic wall on sound. Fluid-solid interfaces were created between the tympanic membrane and air, between the artificial auditory ossicle and air, and between the tympanic vibrating material and air. The contact between these structures and air was set to be frictionless. The TM, artificial auditory ossicle, and tympanic vibrating material were treated as linear-elastic materials, with mechanics parameters shown in Table 2. The material properties of the TM referred to the reported elastic modulus of the tense part of the TM (Volandri et al., 2011). The material properties of the artificial auditory ossicle used the material properties of titanium alloys which was most commonly used as artificial auditory ossicle material. The material properties of the tympanic vibrating material referred to the material properties of silicone. To evaluate the influence of tympanic vibrating material on tympanum vibration, its elastic modulus was set to 0.04, 0.4, and 1 Mpa. The density and Poisson’s ratio of the tympanic vibrating material with different shapes were controlled. The speed of sound in the tympanic membrane, artificial auditory ossicle, and tympanic vibrating material was set to 5,000 m/s.

**TABLE 2:** The material mechanical parameters.

Materials	*E*/MPa	*ν*	*ρ*/g·cm^−3^
Tympanic membrane	30	0.35	1.20
Artificial auditory ossicle	96,000	0.36	4.65
Tympanic vibrating material	0.04, 0.4, and 1	0.35	1.02

*E*: the elastic modulus; *ν*: the Poisson’s ratio; *ρ*: the density.

The contact conditions between the TM and the TORP, as well as the contact between the TORP and tympanic vibrating material, were set to rough and hard. Displacement and rotation of the side surface of the tympanic membrane were limited to simulate the supporting effect of the external auditory canal on the tympanic membrane. The displacement of the bottom surface of the tympanic vibrating material was also restricted to simulate the supporting effect of the promontory on the tympanic vibrating material. According to Thompson (Thompson et al., 2023), the cochlear fluid can be simulated as a spring load on the stapes footplate with a spring constant of 60 N/m. Considering the difference in area between the stapes floor (about 3.2 mm^2^) and the ossicular rod (π * 0.2 mm * 0.2 mm), the spring constant can be set to be 1 N/mm. So, the bottom surface of the artificial auditory ossicle rod was supported by elastic support with a foundation stiffness of 8 N/mm^3^ to simulate the cochlear fluid boundary.

To further substantiate the hearing loss improvement with tympanic vibrating material in the model, the air in the tympanic cavity in Figure 2A was replaced by water to simulate the middle ear vibration conduction with middle ear effusion.

The frequency response curve of the artificial auditory ossicle displacement was extracted. When the stiffness damping coefficient was set to 0.00003, the simulated curve was close to the reported measurement curves. The curves of different tympanic vibrating materials were compared to evaluate the influence of the tympanic vibrating material on the vibration of the tympanum.

### 2.3 LDV measurements to evaluate the effectiveness of the finite element model

**FIGURE 3:**
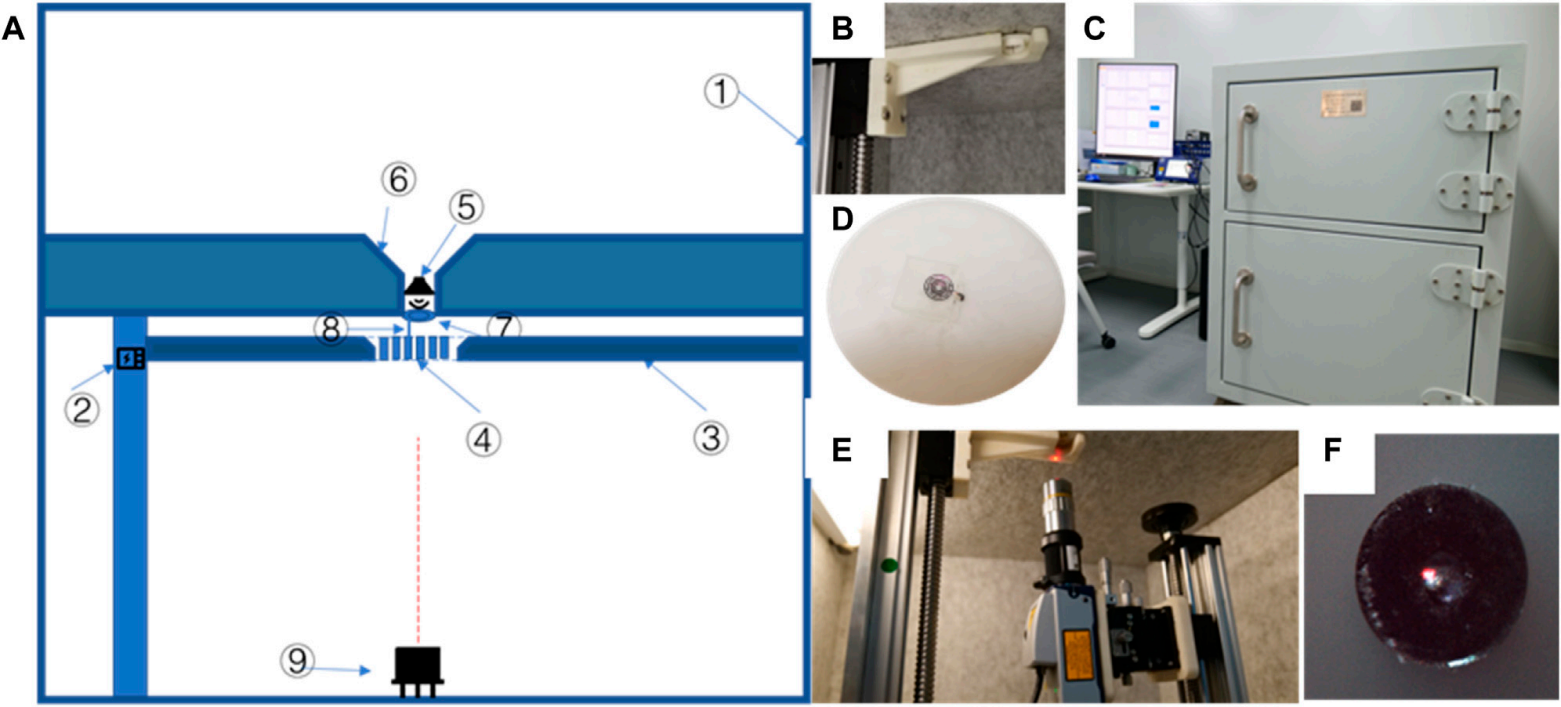
The middle ear Laser Doppler Vibration measurement platform. **(A)** the pattern diagram, ①soundproof box, ②drive motor, ③elevatable platform, ④perforated ossicular placement plate, ⑤amplifier, ⑥external ear model, ⑦tympanic membrane, ⑧TORP artificial auditory ossicle, ⑨Laser Doppler Vibrometer (LDV); **(B)** TORP crossed through the platform inside of the soundproof box; **(C)** The physical image of the whole platform; **(D)** The cooperation method between TORP artificial auditory ossicle and tympanic vibrating material on the perforated ossicular placement plate ④; **(E)** Laser generator of the LDV; **(F)** Laser irradiation to the center of the bottom surface of the TORP under a microscope.

Laser Doppler vibrometer (LDV) is a kind of precision optical instrument that uses laser Doppler effect and interference phenomenon to measure object vibration. Its working principle is that the optical heterodyne detection technology is used to detect the Doppler frequency shift of the scattered laser relative to the incident laser when the object surface is moving, and the electrical signal containing signal optical information is output. The motion information is obtained by signal processing and calculation. As the structure of the middle inner ear is complex and small, the traditional vibration measuring instrument cannot be used in the study of its vibration characteristics. With its unique advantages, LDV can measure the movement of structures of the middle ear and inner ear when coupled with the microscope. LDV has gradually become an important experimental means in otology research and is expected to become a basic equipment for monitoring in otology in the future. To evaluate the effectiveness of the finite element model, we built a Laser Doppler Vibration measurement platform shown in Figure 3A. The measurements were conducted in the soundproof box (Figures 3B,C). A plug-in amplifier was inserted into the external ear, the tympanic membrane was replaced by the temporal muscle fascia, the TORP artificial auditory ossicle, and a 40 kPa solid tympanic vibrating material were placed on the perforated ossicular placement plate and cooperated as Figure 3D. The tympanic vibrating material was processed based on Polydimethylsiloxane (PDMS, C-0030B, A: B = 1:1, Hangzhou Weisichuang Technology Co., Ltd.). The ossicular rod passed through the hole in the perforated ossicular placement plate. Adjust the height of the elevatable platform by controlling the drive motor to make the TORP to be contact with the tympanic membrane. The 90 dB sound was exerted and the Laser Doppler Vibrometer (LDV, VFX-F-110, Polytec, Germany) was used to measure the vibration of the bottom surface of the TORP (Figures 3E,F).

## 3 Results

### 3.1 The validation of sound-structure coupling finite element model

**FIGURE 4:**
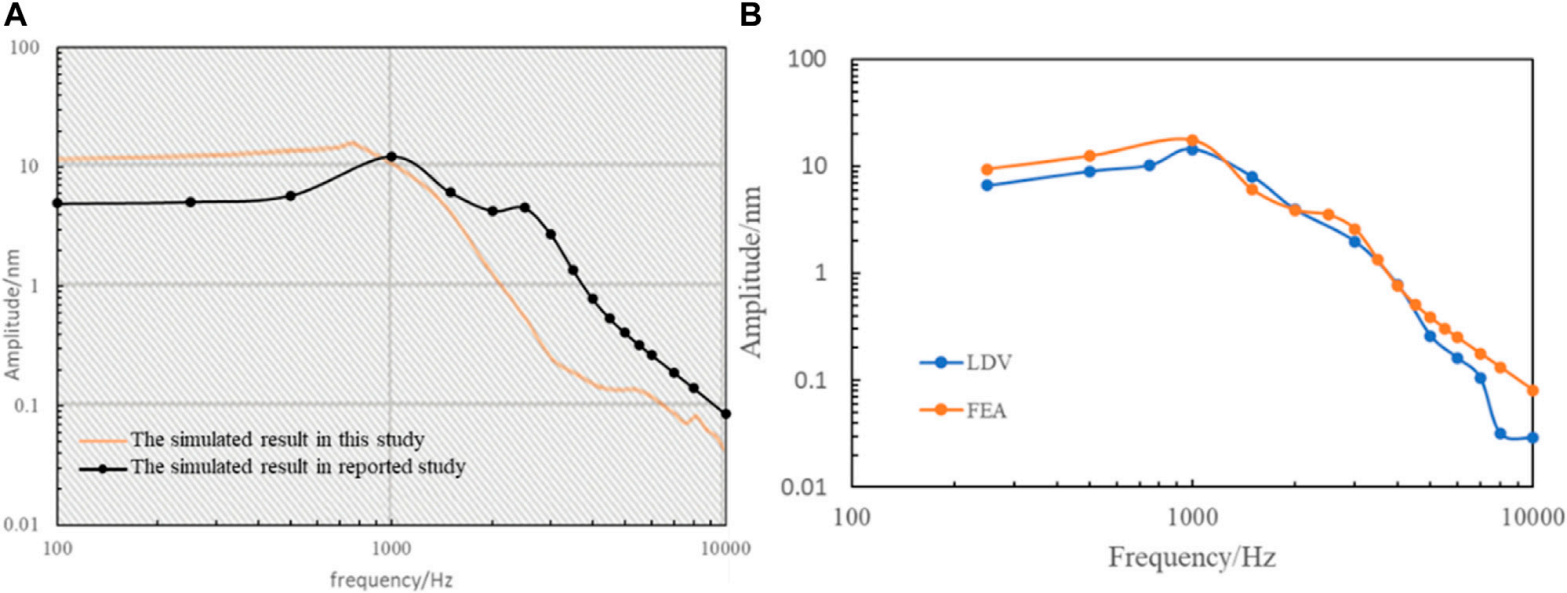
The results of validation of the Finite Element Model. **(A)** Comparation of our simulated frequency response of the artificial auditory ossicle without tympanic vibrating material and the reported results (Hu et al., 2019). **(B)** Comparison of the simulated and LDV measurements of the frequency response of the artificial auditory ossicle with 40 kPa solid tympanic vibrating material.

To validate the effectiveness of the sound-structure coupling finite element model, we extracted the harmonic acoustics result of the normal artificial auditory ossicle without tympanic vibrating material and displayed it in Figure 4. The vibration amplitude of the artificial auditory ossicle increased slowly from 100 Hz to 1,000 Hz and then decreased gradually after 1,000 Hz. The vibration peaked at about 1,000 Hz with an amplitude of 12.30 nm. From Figure 4A, our simulated vibration curve of the artificial auditory ossicle has a similar trend of the frequency response curves and the same order of magnitude of vibration amplitude (Hu et al., 2019) to the stapes footplate vibration curve reported in the references. Besides, the simulated vibration curve of the artificial auditory ossicle has a similar variation trend and magnitude with our LDV measurements (Figure 4B). These results demonstrated that our sound-structure coupling finite element model is reliable and effective.

### 3.2 The harmonic acoustics result of the artificial auditory ossicle with different tympanic vibrating material

**FIGURE 5:**
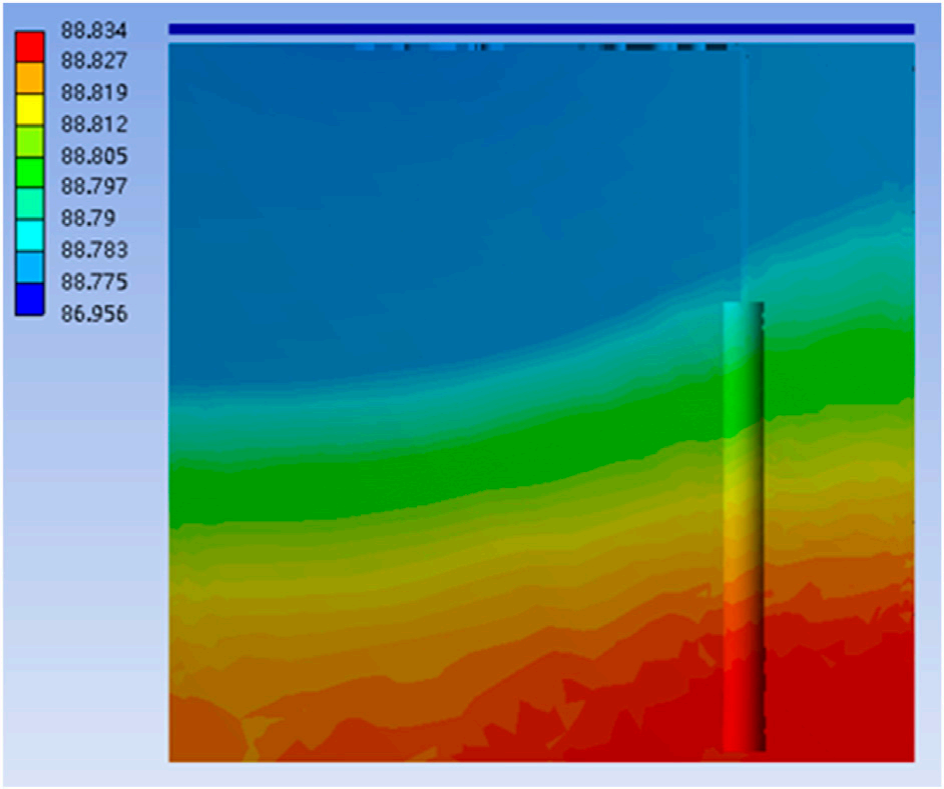
The A-weighted sound pressure level (dBA) in the model without tympanic vibrating material.

Figure 5 shows the A-weighted sound pressure level in the model without tympanic vibrating material. The 90 dB sound pressure made an 87 dB uniform sound field on the surface of the TM, and an 88.8 dB uniform sound field in the tympanic cavity.

**FIGURE 6:**
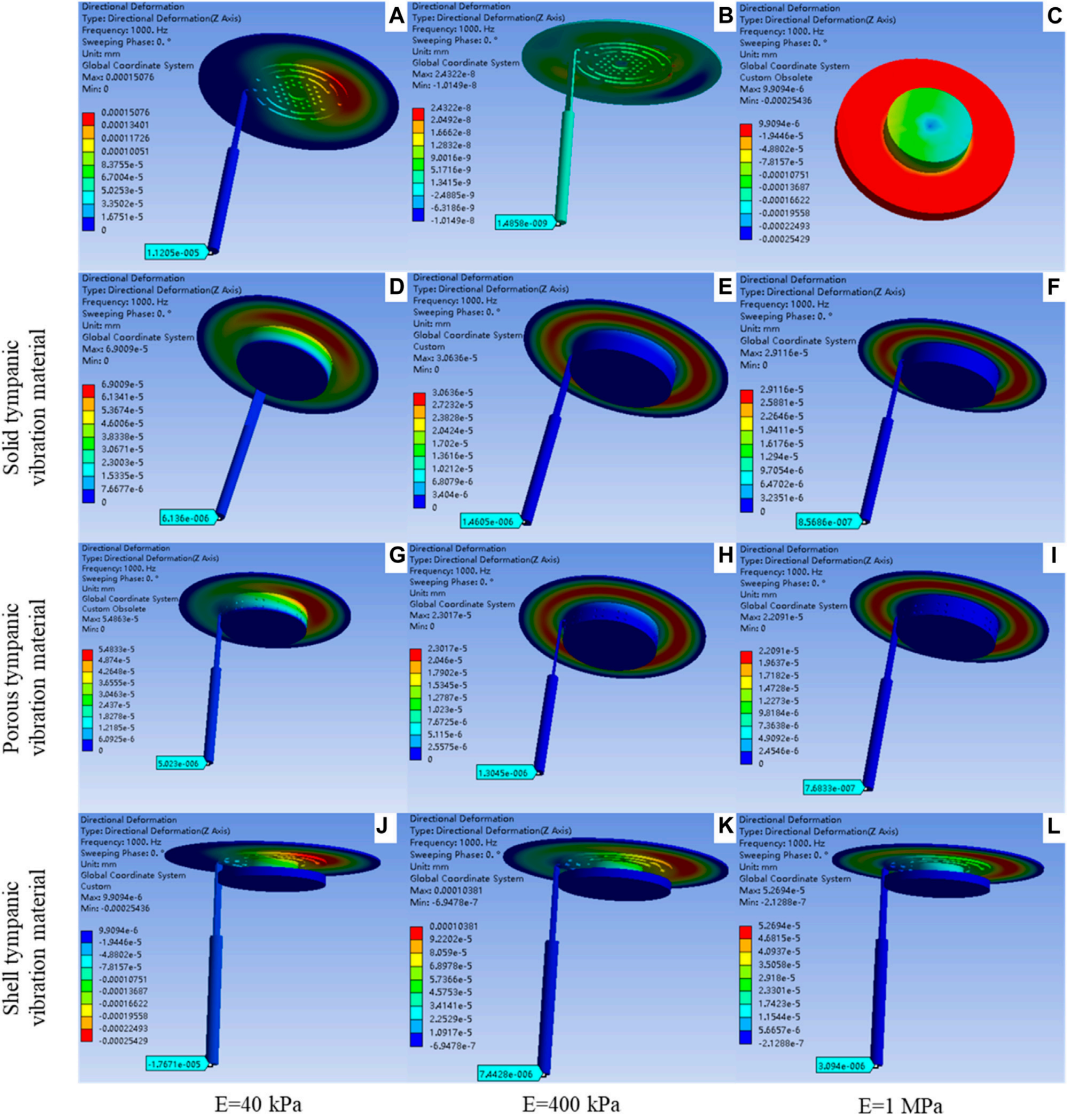
The deformation map of the artificial auditory ossicle and tympanic vibrating material with 1000 Hz sound pressure. **(A)** deformation map of the model without tympanic vibrating material; **(B)** Deformation map of the model with water tympanic cavity; **(C)** Deformation map of the 40 kPa shell tympanic vibrating material; **(D–F)** Deformation map of the model with solid tympanic vibrating material [40 kPa **(D)**, 40 kPa **(E)**, 1 MPa **(F)**]; **(G–I)** Deformation map of the model with porous tympanic vibrating material [40 kPa **(G)**, 40 kPa **(H)**, 1 MPa **(I)**]; **(J–L)** Deformation map of the model with shell tympanic vibrating material [40 kPa **(J)**, 40 kPa **(K)**, 1 MPa **(L)**].

Results of finite element analysis showed that the tympanic vibrating material vibrates under sound pressure, which provides space for the vibration of the ossicle and ensures the vibration of the ossicles (Figure 6). Figure 6 shows the deformation map of the artificial auditory ossicle with shell tympanic vibrating material. These results demonstrated the possibility of hearing rehabilitation by tympanic vibrating material.

**FIGURE 7:**
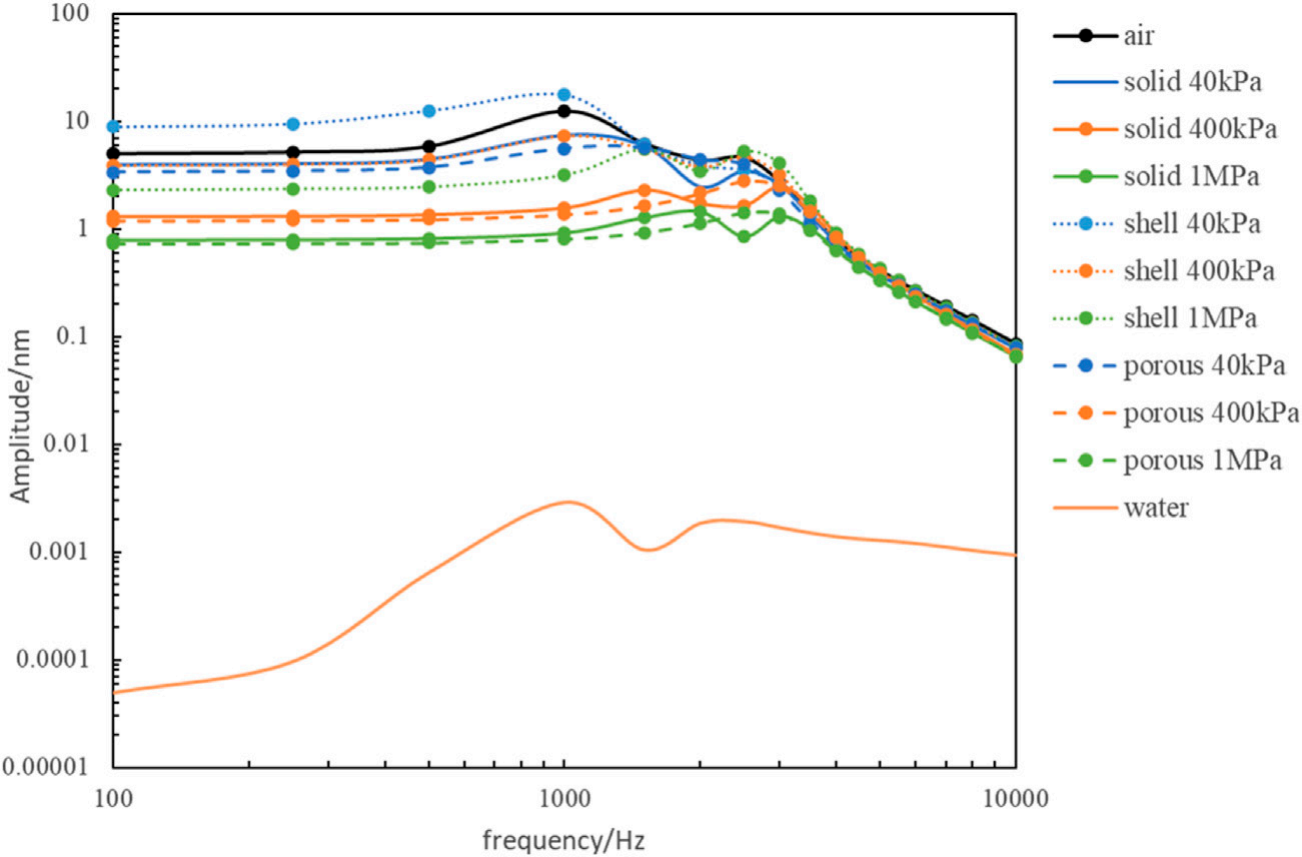
The comparison of the frequency response of the artificial auditory ossicle with different tympanic vibrating material.

Figure 7 shows that the frequency response curve of artificial auditory ossicles with solid, porous, and shell tympanic vibrating materials has similar variation trends compared to that in the air. Vibration amplitude decreased with increasing elastic modulus of tympanic vibrating material, and loss of vibration amplitude was more significant at frequencies lower than 3,000 Hz. Shell tympanic vibrating material had a better vibration conduction effect compared to solid tympanic vibrating material and porous tympanic vibrating material with the same elastic modulus. From Figure 7 we can also find that implantation of tympanic vibrating material can significantly improve the hearing loss caused by ear effusion.

**FIGURE 8:**
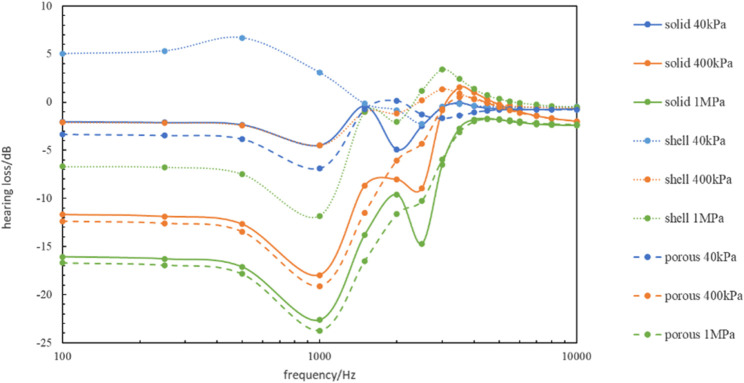
The hearing loss with different tympanic vibrating material compared to the normal model.

Comparing the vibration of the artificial auditory ossicle with different tympanic vibrating material with its vibration in the air, we can get hearing loss (Figure 8). From Figure 8 we can find the implantation of 40 kPa-shell tympanic vibrating material had the lowest hearing loss of less than 5 dB, and the hearing loss with 1 MPa-porous tympanic vibrating material was largest and less than 25 dB. All of the hearing loss was within the clinically acceptable range, which suggested that these tympanum vibrating materials can achieve the purpose of hearing reconstruction.

**FIGURE 9:**
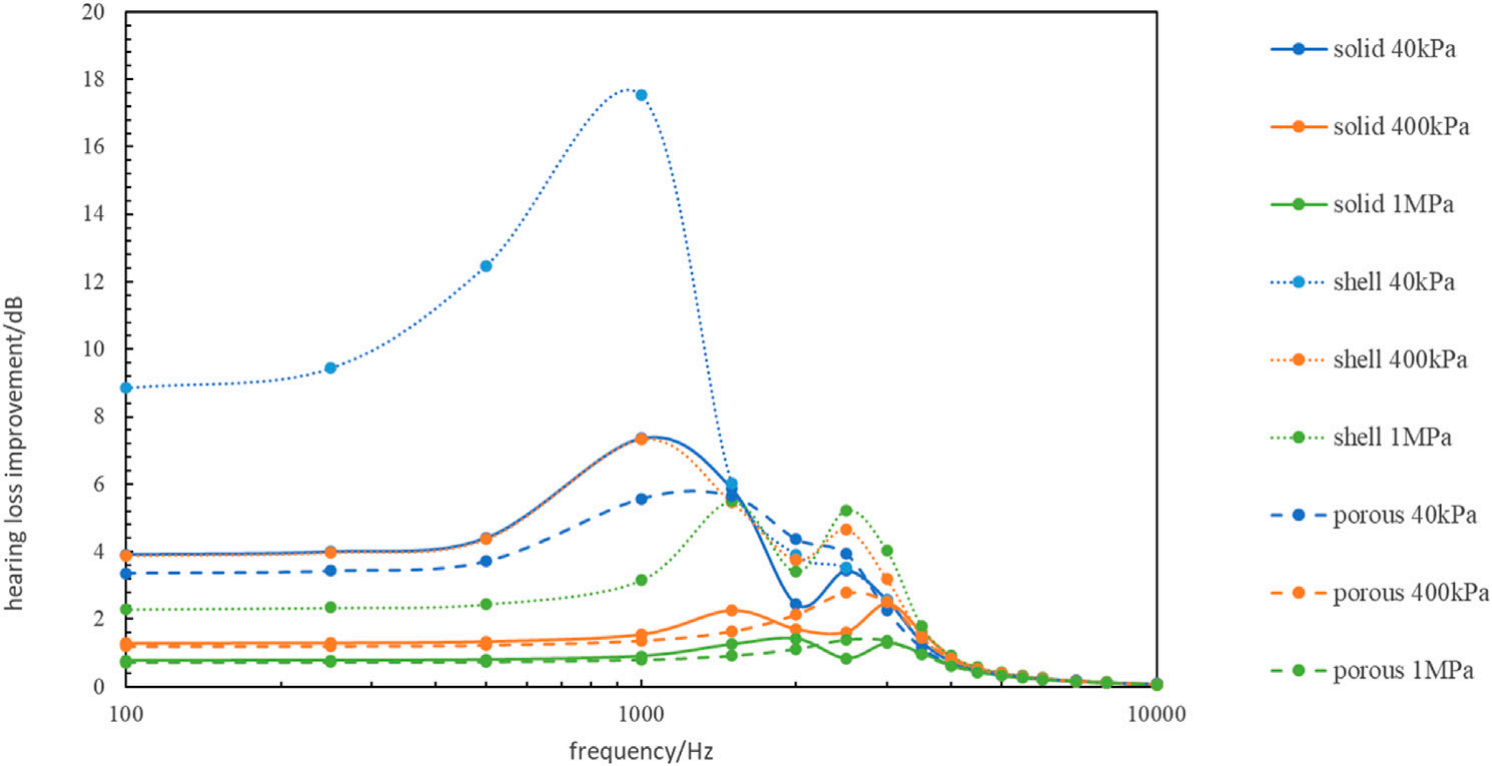
The hearing loss improvement with different tympanic vibrating material compared to the model with middle ear effusion.

Comparing the vibration of the artificial auditory ossicle with different tympanic vibrating materials with its vibration in the water, we can get the hearing loss improvement (Figure 9). From Figure 9 we can find the implantation of 40 kPa-shell tympanic vibrating material can significantly improve the hearing loss caused by ear effusion with hearing loss improvement 8–20 dB. Other tympanic vibrating material implantation can also improve the hearing loss caused by ear effusion 0–8 dB.

## 4 Discussion

The problem of hearing reconstruction in patients with tympanic adhesion, whose air cavity cannot be reconstructed, is clinically significant. Adhesive tissue inhibits normal vibration, leading to conductive hearing loss. To address this, we proposed a hypothesis in this study that implanting vibrating materials into the tympanum can prevent adhesive tissue and provide sufficient space for ossicular vibration. Finite element analysis validated the hypothesis, indicating that hearing conduction may be possible independent of the tympanic air cavity.

According to our hypothesis, finding suitable vibration materials is the key to hearing reconstruction independent of the tympanic air cavity. The finite element method is a convenient, low-cost, and repeatable method for design and optimization, which is widely used in mechanical design due to its advantages of easily changing the structure and material parameters of models. The finite element method is also widely used in the biomechanics of the middle ear (Kiryk et al., 2018; Gil Mun et al., 2019; Mocanu et al., 2021). This study developed a sound-structure coupling finite element model to evaluate the effectiveness of implanting vibrating materials into the tympanum. Results showed that the tympanic vibrating material vibrates under sound pressure, which provides space for the vibration of the ossicle and ensures the vibration of the ossicles which demonstrates the possibility of hearing rehabilitation by tympanic vibrating material.

The vibration amplitude of the stapes footplate is a commonly used parameter for middle ear mechanics researchers and an important indicator for assessing hearing ability (International, 2005; Gladine and Dirckx, 2018; Yao et al., 2018; Borkowski et al., 2019). It reflects the extent to which sound waves are transmitted through the middle ear and ultimately to the inner ear, which is critical for normal hearing function. Therefore, the vibration amplitude of the stapes footplate is extracted in this study to evaluate the influence of the tympanic vibrating material on the vibration of the tympanum. The simulated vibration amplitude of the artificial auditory ossicle increased slowly from 100 Hz to 1,000 Hz and then decreased gradually after 1,000 Hz. The simulated vibration curve of the artificial auditory ossicle has a similar variation trend and magnitude with our LDV measurements. Besides, our simulated vibration curve of the artificial auditory ossicle has a similar variation trend and magnitude (Hu et al., 2019) to the stapes footplate vibration curve reported in the references. In comparison with the stapes footplate vibration curve measured by the Laser Doppler Vibrometer reported in the references, our simulated vibration curve of the artificial auditory ossicle has a similar variation trend and magnitude (Yao et al., 2018; Hu et al., 2019), which demonstrates that our sound-structure coupling finite element model is reliable and effective.

The structure and physical parameters of tympanic vibrating material are important indicators that affect their vibration transmission effect. In this study, three structures of tympanic vibrating material were designed, and the results showed that the shell-like structure had a better vibration conduction effect compared to solid or porous materials. Too-hard materials can affect the vibration of the tympanum and ossicles, while too-soft materials make it difficult to support the tympanum and prevent adhesion. In this study, tympanic vibrating material with the elastic modulus of 0.04, 0.4, and 1 MPa was explored to evaluate their vibration conduction function. Results showed the vibration amplitude decreases with the increasing elastic modulus of tympanic vibrating material.

Among all of the tympanic vibrating material in this study, 40 kPa-shell tympanic vibrating material had the lowest hearing loss (less than 5 dB) compared with normal subjects. Even the 1 MPa-porous tympanic vibrating material which had the worst hearing conduction effect results in a hearing loss of less than 25 dB. All of the hearing losses were within the clinically acceptable range. Comparing the vibration of the artificial auditory ossicle with different tympanic vibrating materials with its vibration in the water, we can find the implantation of tympanic vibrating material can significantly improve the hearing loss caused by ear effusion with hearing loss improvement 0–20 dB. These results showed that the tympanic vibrating material could effectively improve the hearing of patients with tympanic adhesion.

The silicone material is a commonly used material for human implantation (Larena-Avellaneda et al., 2008; Goldfinger et al., 2014) with good biocompatibility. The elastic modulus of silicone may adjusted from several kPa to hundreds of MPa (Vlassov et al., 2018). The biocompatibility and adjustable mechanical properties make the silicone a possible tympanic vibrating material. The clinical significance of these findings is significant, particularly for patients with severe tympanic adhesion who may have limited options for treatment. Current methods such as tympanoplasty and bone-guided hearing aids may be ineffective in these cases, and the use of vibrating materials offers a potentially more convenient and effective solution. Additionally, the ability to customize the size and shape of the vibrating materials using 3D printing technology offers the potential for more personalized treatment options and improved outcomes for patients.

Innovatively, this study provides a new solution for hearing reconstruction in patients with severe tympanic adhesion by using tympanic vibrating materials. This technique offers a potentially more convenient and effective solution compared to traditional methods such as tympanoplasty and bone-guided hearing aids. One limitation of this study is that it used a simplified model of the middle ear, which may not fully represent the complex anatomy and physiology of the human middle ear. The normal vibration amplitude of the stapes footplate simulated in this study has a similar variation trend and magnitude with the reported results (Yao et al., 2018; Hu et al., 2019), which demonstrates that our sound-structure coupling finite element model is reliable and effective. A simplified model provides a low-cost method to evaluate the function of tympanic vibrating material rapidly and effectively.

Further research is needed to validate these findings and optimize the design of tympanic vibrating material for hearing reconstruction. On the one hand, the function of tympanic vibrating material can be evaluated by temporal bone implantation experiments and laser Doppler vibration measurement technology. On the other hand, the material, structure, and mechanical properties of tympanic vibrating material may be optimized to obtain safe and long-term effective tympanic vibrating material.

## 5 Conclusion

In conclusion, finite element analysis results showed that material replacing the tympanic air cavity with tympanic vibrating material is feasible. The findings of this study suggest that implanting vibrating materials into the tympanum may provide a promising solution for hearing reconstruction in patients with severe tympanic adhesion. While further research is needed to fully evaluate the safety and efficacy of this approach, these results represent an important step forward in the development of new treatments for hearing loss.

## Data Availability

The original contributions presented in the study are included in the article/Supplementary Material, further inquiries can be directed to the corresponding authors.
